# Cardiomyopathies and Adrenal Diseases

**DOI:** 10.3390/ijms21145047

**Published:** 2020-07-17

**Authors:** Luigi Petramala, Antonio Concistrè, Federica Olmati, Vincenza Saracino, Cristina Chimenti, Andrea Frustaci, Matteo A. Russo, Claudio Letizia

**Affiliations:** 1 Department of Translational and Precision Medicine, Sapienza University of Rome, 00185 Roma, Italy; luigi.petramala@uniroma1.it (L.P.); antonio.concistre@uniroma1.it (A.C.); federica.kolmati@gmail.com (F.O.); vincenza.saracino@ymail.com (V.S.); 2 Clinical Internal, Anesthesiological and Cardiovascular Sciences, Sapienza University of Rome, 00185 Roma, Italy; cristina.chimenti@uniroma1.it (C.C.); biocard@inmi.it (A.F.); 3 MEBIC Consortium, San Raffaele Open University, and IRCCS San Raffaele Pisana, 00185 Rome, Italy; matteoantoniorusso44@gmail.com

**Keywords:** cardiomyopathy, endomyocardial biopsy, primary aldosteronism, Cushing’s syndrome, pheochromocytoma, adrenal disease

## Abstract

Cardiomyopathies are myocardial disorders in which heart muscle is structurally and/or functionally abnormal. Previously, structural cardiomyocyte disorders due to adrenal diseases, such as hyperaldosteronism, hypercortisolism, and hypercatecholaminism, were misunderstood, and endomyocardial biopsy (EMB) was not performed because was considered dangerous and too invasive. Recent data confirm that, if performed in experienced centers, EMB is a safe technique and gives precious information about physiopathological processes implied in clinical abnormalities in patients with different systemic disturbances. In this review, we illustrate the most important features in patients affected by primary aldosteronism (PA), Cushing’s syndrome (CS), and pheochromocytoma (PHEO). Then, we critically describe microscopic and ultrastructural aspects that have emerged from the newest EMB studies. In PA, the autonomous hypersecretion of aldosterone induces the alteration of ion and water homeostasis, intracellular vacuolization, and swelling; interstitial oedema could be a peculiar feature of myocardial toxicity. In CS, cardiomyocyte hypertrophy and myofibrillolysis could be related to higher expression of atrogin-1. Finally, in PHEO, the hypercontraction of myofilaments with the formation of contraction bands and occasional cellular necrosis has been observed. We expect to clear the role of EMB in patients with cardiomyopathies and adrenal disease, and we believe EMB is a valid tool to implement new management and therapies.

## 1. Introduction

Cardiomyopathies (CMPs) include a variety of myocardial disorders appearing with various structural and functional phenotypes. CMPs are divided into two major groups based on the predominant organ involvement. Primary CMPs (genetic, nongenetic, acquired) are specifically or predominantly confined to the heart muscle. Secondary CMPs are characterized by pathological myocardial involvement during several generalized systemic disorders [1].

CMP usually manifests with cardiac failure, which can be mechanic (such as diastolic or systolic dysfunction) or due to electrical disorders (i.e., long QT syndrome and Brugada syndrome).

In latest the Consensus Statement of the American Heart Association (AHA) [1], any pathological myocardial dysfunction derived as a consequence of primary cardiovascular disturbances is not included in the definition of CMPs. Those pathologies include valvular heart diseases, arterial hypertension, congenital heart disease, and ischemic heart disease; other conditions not included are metastatic and primary heart cancers.

Besides, physicians admit that a distinction between primary and secondary cardiomyopathy is purely arbitrary, because many of these cardiac abnormalities are associated with systemic disorders, for example, amyloidosis or glycogen storage diseases.

Interestingly, CMPs due to adrenal hormone excess are not included in the classification of the AHA Consensus Statement [1]. In spite of these observations, in subjects with secondary arterial hypertension, the myocardial involvement has never been systematically studied with precise methodology.

Endomyocardial biopsy (EMB) has been demonstrated to be a useful tool in disclosing the definitive etiology of cardiomyopathy [2]. However, the use of left ventricular (LV) EMB was discouraged because it was considered a risky procedure, since so many latent structural abnormalities were misdiagnosed. Using a very large database of EMBs from 4221 patients, Chimenti and Frustaci demonstrated that EMB is a safe procedure with very low transient complications and a very important diagnostic contribution, if performed in centers with a high expertise of its trained physicians and a large number of procedures [3].

Microscopic and ultrastructural cardiac alterations have still not been specifically evaluated in patients suffering from aldosterone, cortisol, or catecholamine excess. It is a matter of debate if patients affected by hormonal excess due to primary adrenal disorders, with echocardiographic evidence of altered indexes with a preserved or reduced ejection fraction, have to be treated by these procedures.

Herein, we describe the most prominent features of CMPs due to adrenal diseases, from in vitro and in vivo research, to current and clinical evidence obtained by echocardiographic examinations and cardiac magnetic resonance. We browsed a bibliography of research of the current knowledge regarding the excess of aldosterone, cortisol, and catecholamine-induced CMPs. Finally, we illustrate data obtained by EMBs, which firstly reveal new mechanisms and pathways implied in the pathogenesis of myocardial structural abnormalities. Those features would be useful in adopting and addressing an appropriate strategy for providing new therapies and management for cardiovascular complications in the case of inappropriate hormone production. Moreover, these specific conditions of endogenous hormonal excess (aldosterone, cortisol, and catecholamines) can represent a human model to study several CMPs observed during several systemic diseases or drug-induced side effects.

## 2. Aldosterone-Induced Cardiomyopathies

Primary aldosteronism (PA) is a condition characterized by inappropriate aldosterone secretion by the adrenal gland, not related to physiological condition (i.e., electrolyte balance, blood volume) [4]. The prevalence of PA accounts 10–11% of patients with arterial hypertension, and up to 22–30% of patients with resistant hypertension [5]. Common causes of PA are unilateral aldosterone-producing adenoma (APA) of the adrenal gland and unilateral or bilateral adrenal hyperplasia (IHA).

The relationship between heart failure and myocardial infarction is deeply understood, whereas elevated aldosterone concentrations can contribute to the development of cardiovascular remodeling through several mechanisms beyond blood pressure levels. Thus, aldosterone overproduction generates other deleterious effects in vascular and cardiac remodeling, favoring collagen deposition and cardiovascular fibrosis, endothelial dysfunction, perivascular inflammatory and fibrotic lesions, oxidative stress, and renal dysfunction [6,7,8,9,10]. 

In an interesting review conducted by Monticone et al., an increased rate of cardiovascular events, such as atrial fibrillation, stroke, and myocardial infarction, has been found in PA patients, compared with patients with essential hypertension (EH), suggesting direct myocardial damage caused by aldosterone overproduction [11].

It is well known how aldosterone exerts its damage by genomic and nongenomic effects. Genomic effects are expressed by aldosterone binding to intracellular receptors followed by the transcription of specific genes, implicated in cardiovascular homeostasis, vascular tone regulation, and hydro-electrolyte balance at the level of epithelial tissues. On the other hand, nongenomic effects are more specifically expressed in tissues and nonepithelial structures of the heart, vessels, and kidney [12].

The physiopathology of PA-mediated hypertension includes plasma volume expansion through sodium and fluid retention and vasoconstriction from potassium depletion. Moreover, aldosterone induces higher oxidative stress and decreased nitric oxide (NO) bioavailability, favoring reduced endothelium-dependent relaxation [10]. Furthermore, aldosterone-mediated perivascular fibrosis reduces vascular compliance [13].

An excess of aldosterone is also implicated in the development of metabolic syndrome and insulin resistance, affecting the balance of several adipokines secreted by visceral adipose tissue, such as leptin, adiponectin, and resistin [14]. Moreover, aldosterone interacts with epicardial fat, an active metabolic tissue that is implied in the increase of LV mass in PA patients [15].

In experimental rat models, aldosterone infusion induces cardiac hypertrophy, as documented through echocardiography. Moreover, hormone excess determines macrophage infiltration in the cardiac interstitium and focal inflammatory lesions. These findings support the thesis that aldosterone by itself sustains cardiac hypertrophy and severe inflammatory responses in the heart, independently of salt loading or nephrectomy [16].

Although clinical and experimental studies have suggested a remarkable role for aldosterone in modulating myocardial hypertrophy, it is difficult to distinguish between direct action on the growth/differentiation of myocytes and the indirect effects due to mechanical overload. Okoshi et al., have evaluated the possible influence of aldosterone in the development of myocyte hypertrophy in low-density and serum-free cultures of ventricular myocytes in neonatal rats [17]. As a result, aldosterone excess increased protein incorporation (+27%) and myocyte surface area with respect to the vehicle control (+29%). This phenomenon was associated with increased mRNA levels of the atrial natriuretic factor and alpha- and beta-myosin heavy chains. Moreover, a mineralocorticoid receptor blocker (i.e., spironolactone) was able to prevent this process. In conclusion, aldosterone directly stimulates hypertrophy in ventricular myocytes of neonatal rats, through the direct activation of mineralocorticoid receptors and related transcriptional factors. Furthermore, aldosterone also promotes collagen deposition, the stimulation of inflammatory cells, and fibroblast proliferation.

Therefore, the hyperstimulation of cardiovascular aldosterone receptors in humans is associated with arterial hypertension, myocardial hypertrophy with sarcomere gene overexpression, left ventricular systo-diastolic dysfunction, and fibrosis.

A study conducted by Matsumura et al., aimed to determine the role of aldosterone in LV geometry in human subjects [18], evaluating 25 PA patients with APA, 29 patients affected by renovascular hypertension (RVH), and 29 EH patients, matched for blood pressure behaviors (by 24-h systolic and diastolic BP). Adjusted LV mass index (for age, sex, mean 24-h systolic BP, mean 24-h pulse rate, body mass index, and duration of hypertension) significantly increased in the PA and RVH groups compared with EH patients (150.2 ± 7.7 g/m^2^, 142.3 ± 7.2 g/m^2^, and 115.2 ± 7.2 g/m^2^, respectively). Vascular damage related to hypertension, such as proteinuria and hypertensive retinopathy, was more pronounced in RVH patients, whereas LV hypertrophy was higher in PA patients. These results confirm that aldosterone might induce LV hypertrophy, in human beings as well as in experimental animals, and that angiotensin II and aldosterone might differentially participate in causing hypertensive vascular organ damage.

Galetta et al., conducted an observational study in 23 PA patients, 24 EH patients, and 15 normotensive controls (NS), evaluating myocardial properties with conventional echocardiography and tissue doppler (TDI) analysis. In addition, they performed ultrasonic tissue characterization by the cyclic variation of integrated backscatter (CVIBS). PA patients showed a septal and posterior wall CVIBS that was significantly lower than EH patients and NS; thus, the authors concluded that PA patients showed myocardial wall remodeling characterized by increased myocardial fibrosis and early left ventricular function abnormalities [19].

Previously, Rossi et al., consecutively evaluated 34 PA patients and 34 EH subjects, matched for age, body mass index, BP, and the duration of hypertension [20]. It was found that significantly more PA patients with respect to EH patients had LV hypertrophy or concentric remodeling (50% versus 15%, χ2 = 11.97, *p* = 0.007); both the E wave flow velocity integral (1063 ± 65 versus 1323 ± 78, *p* = 0.013) and the integral E/A ratio were lower (0.91 ± 0.05 versus 1.25 ± 0.08, *p* < 0.001), and the atrial contribution to LV filling was higher (53.3 ± 1.5% versus 45.5 ± 1.3%, *p*< 0.001) in PA patients compared to EH patients. Moreover, in PA patients, LV mass was higher compared to EH patients, although there were comparable BP values in both groups. After one-year of follow-up, significant decreases in LV wall thickness and mass were observed in PA patients if surgically treated (adrenal removal due to aldosterone-producing adenoma); conversely, reverse remodeling was not observed in the group that was pharmacologically treated. Thus, in PA patients, aldosterone excess is associated with both increased LV mass and significant changes in LV diastolic filling, though these structural and functional changes appear to be reversible after the removal of aldosterone excess.

Furthermore, Cesari et al., observed an excessive LV hypertrophy and diastolic dysfunction in PA patients, as well as in the conditions characterized by secondary aldosteronism (SA) (i.e., liver cirrhosis) but not associated with arterial hypertension [21]. The authors investigated cardiac modifications in 262 patients with PA and 117 patients with SA due to liver cirrhosis, measuring echocardiographic parameters, including TDI and strain rate analysis. SA and PA patients showed markedly elevated aldosterone levels but different behaviors of plasma renin activity. Compared with PA patients, SA patients showed a higher heart rate, lower BP, and lower vascular resistance values. Both PA and SA patients showed increased LV diameters, LV volumes, stroke volume, stroke work, and septal peak systolic tissue velocity, as well as higher LV hypertrophy (61 and 39%, respectively) and diastolic dysfunction (35% and 36%, respectively). This study concluded that PA and SA are associated with LV enlargement and a high prevalence of LV hypertrophy and diastolic dysfunction, but a subclinical systolic dysfunction was evident only in PA patients. In SA patients, the high rate of LV hypertrophy, despite low peripheral resistances and low to normal BP, could be accounted for by the high renin and aldosterone values and volume overload.

Freel et al., have evaluated the effects of aldosterone excess on myocardial structure in human patients affected by PA and EH using contrast-enhanced cardiac magnetic resonance imaging [22]. They recruited 27 PA and 54 EH patients, observing a significant increase in non-infarct late gadolinium enhancement in PA patients (70%) when compared with EH subjects (13%; *p* < 0.0001). Moreover, pulse wave velocity, superoxide, and C-reactive protein levels were significantly higher in PA patients.

**Figure 1:**
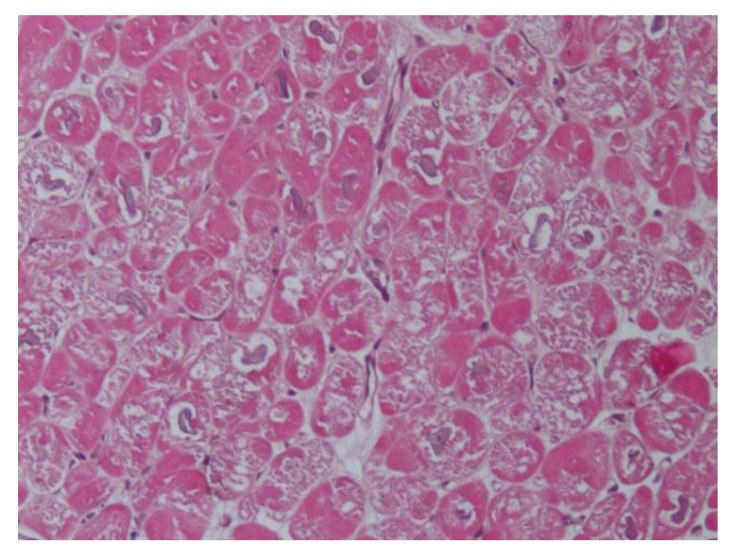
Hematoxylin and eosin staining (200×) shows enlarged and vacuolated myocardiocytes due to water accumulation.

Recently, using EMB, we have reported histological and ultrastructural myocardial changes in four PA patients (affected by aldosterone-producing adrenal adenoma) admitted because of worsening dyspnea (NYHA class 2–3) [23]. In one patient, we re-evaluated the biopsy 12 months after the surgical removal of APA, aldosterone normalization, and the recovery of cardiac function. In the EMB series, the increased volume of cardiomyocytes containing large intracellular vacuoles was evident (Figure 1). Furthermore, the expression of myocardial aldosterone receptors and aquaporin-1 and 4 were visualized by using a mineralocorticoid receptor monoclonal antibody, anti-Aqp1, and anti-Aqp4.

In the ultrastructural examination, vacuoles were filled with an electron-clear homogeneous content, suggesting ion and water accumulation. Vacuoles were diffusely present inside the cytoplasm and were enclosed by a single membrane, associated with the sarcoplasmic reticulum and Golgi apparatus, suggesting that they may originate upon the dilatation of cisternae of the sarcoplasmic reticulum and Golgi apparatus. Mitochondria and lysosomes appeared to be electron clear, likely due to a dilution of their matrix upon ion and water increase (swelling). The interstitial space was widened because of both interstitial edema (amorphous electron-clear spaces) and fibrosis. Finally, several areas of myofibrillolysis and numerous autophagosomes were observed as a result of myocardiocyte damage.

A western blot for the mineralocorticoid receptor and aquaporin-1 showed, respectively, a 2.8-fold and a 3-fold increase in protein expression, compared to EH patients. The overexpression and intracellular localization of aquaporin-1 suggest that this channel might be responsible for abnormal water movement among intracellular compartments.

As a main and new clinical implication for the understanding of aldosterone cardiomyopathy, this study showed that aldosterone induces an alteration in ion and water homeostasis, through abnormal water compartmentation, vacuolar degeneration, and interstitial edema. Overall, this study shows the specific histological findings associated with aldosterone-induced cardiomyopathy and underlines the role of EMB in the diagnosis and management of the disease.

The astounding findings of cardiomyocyte edema and vacuolar degeneration reflect the already known concept that excessive aldosterone determines plasma volume expansion from sodium and fluid retention [13]. This mechanism is likely to be implicated in cardiac failure in the case of hypertensive heart disease in PA.

Intracellular vacuolization and swelling could be peculiar features of specific aldosterone-mediated myocardial toxicity. These preliminary results are in agreement with clinical and subcellular damage observed before: an increase in aldosterone receptors and aquaporin-1, electrolyte alterations, vacuole formation, mitochondrial and cytosol swelling, and hypertrophic sarcomeric disarray, which are all converging features that alter contractility and relaxation properties and provoke long-term myocardiocyte damage and myofibrillolysis.

The final process of aldosterone cardiac damage results in fibrotic repair, and consequently progressive functional deterioration that is irreversible, even in the case of the surgical removal of inappropriate aldosterone excess.

The recommended treatment for PA is unilateral laparoscopic adrenalectomy in patients with proven unilateral PA, or medical treatment with mineralocorticoid receptor blockades in patients not eligible for surgery or with bilateral adrenal hyperplasia [4].

From the publication of the results of the Randomized Aldactone Evaluation Study (RALES) and the Eplerenone Post-Acute Myocardial Infarction Heart Failure Efficacy and Survival Study (EPHESUS), aldosterone blockade was added to the clinical guidelines for the management of chronic heart failure [24,25]. An aldosterone blockade induces a reduction in left ventricle (LV) remodeling and collagen deposition, an improvement in endothelial function, decreased inflammation, and increased myocardial perfusion [26,27,28].

Catena et al., evaluated long-term cardiac structural and functional evolution in patients with PA after surgical or medical treatment [29], comparing echocardiographic measurements of PA patients with an EH group. PA patients had more significant LV mass, more prevalent LV hypertrophy, lower early/late-wave diastolic filling velocity ratios, and longer deceleration times than EH patients. After one-year of follow-up, left ventricular mass decreased significantly only in adrenalectomized patients, whereas in patients treated with spironolactone, changes in left ventricular mass were significant at the end of the study (average follow-up of 6.4 years). Moreover, changes in BP and pretreatment plasma aldosterone were independent predictors of LV mass. This study confirms that, in PA patients, surgical and medical treatments are useful in reducing LV mass and decreasing BP and plasma aldosterone levels, the latter showing a predictive role in terms of therapeutic response.

Afterwards, as a confirmation of this evidence, the objective of Lin et al., was to investigate myocardial fibrosis in PA patients and possible recovery after surgery [30], evaluating in 20 APA patients who received adrenalectomy, matched with 20 EH patients, the plasma carboxyterminal propeptide procollagen type 1 (PICP) and echocardiography with ultrasonic tissue characterization of the CVIBS at baseline and one-year of follow-up after the surgical procedure. APA patients had higher LV mass index, significantly lower CVIBS and higher PICP levels than EH patients at baseline. Moreover, the CVIBS significantly increased, while plasma ICP levels decreased, one year after adrenalectomy. According to these findings, the authors concluded that increased collagen content in the myocardium of APA patients could be reversible after adrenalectomy. In previous work, Frustaci et al. showed that, after the surgical removal of APA and the subsequent normalization of aldosterone levels, cardiomyocytes appeared smaller with the disappearance of vacuoles in the cytoplasm, and myofibrillolysis and autophagocytosis were no longer visible; nevertheless, fibrosis remained unchanged in comparative morphometric analysis [23].

In conclusion, PA cardiomyopathy is a reversible entity characterized by an overexpression of myocardial aldosterone receptors and aquaporin-1 channels, with cardiomyocyte and microvascular water accumulation, maladaptive hypertrophy, areas of myofibrillolysis, and the impairment of myocyte relaxation and contraction.

## 3. Cortisol-Induced Cardiomyopathies

Hypercortisolism is a condition clinically characterized by hypertension, central obesity, insulin resistance, dyslipidemia, and alterations in coagulation and platelet function [31].

Hypertension is found in about 80% of patients with Cushing’s syndrome (CS), characterized by changes in the regulation of plasma volume, systemic vascular resistance, and vasodilation [32].

Furthermore, patients with hypercortisolism often have impaired fasting glucose, impaired glucose tolerance, hyperinsulinemia, insulin resistance and/or diabetes mellitus [33], decreased HDL concentration, and increased triglycerides [32]. In 2003, Muiesan et al., conducted a study on 42 consecutive patients with newly diagnosed CS, matched for age, gender, and BP, with 42 control subjects [34], evaluating LV mass index and relative wall thickness (RWT) by echocardiography. RWT was significantly greater in CS patients than in controls, and LV hypertrophy and concentric remodeling were respectively observed in 10 (24%) and 26 (62%) CS patients. A significantly higher frequency of concentric remodeling (normal LV mass and concentric geometry) was concomitant in patients with CS than in controls, accurately matched not only for demographic characteristics but also for BP values, previous antihypertensive therapy, and duration. So, the authors concluded that cardiac structural changes are associated with reduced mid-wall systolic performance and with diastolic dysfunction, explaining the high risk of cardiovascular events observed in these patients.

Dilatative cardiomyopathy is rarely associated with CS and only a few cases are reported in the literature [35]. Because of its rarity, the histological changes and molecular pathways involved in its pathophysiological features are poorly understood. Frustaci et al., reported the case of a 63-year-old woman affected by CS due to cortisol-producing adrenal adenoma and coexisting dilatative cardiomyopathy and severe hypokinesia (ejection fraction less than 24%) [36].

Echocardiographic parameters (LV end-diastolic diameter, ejection fraction, and maximal wall thickness) and EMB findings were evaluated at baseline and after one year of cortisol normalization due to adrenalectomy. At diagnosis, cell swelling, myofibrillolysis, and the partial disorganization of sarcomeres were clear, whereas after one-year of follow-up there was a sustained recovery of cell volume, cytosol density, and sarcomeric organization. In addition, myocardial atrogin-1 was evaluated at presentation and after one-year of follow-up.

Atrogin-1 is a protein with enhanced expression during the muscle atrophy process. It has been shown how mRNA for atrogin-1 is abnormally expressed in all types of atrophy studied and its expression increases during muscle weight loss [37]. It is well known how muscle atrophy occurs during fasting, or in other pathological conditions such as cancer, diabetes mellitus, AIDS, sepsis, and hypercortisolism (endogenous or exogenous).

In Frustaci et al.’s study, the expression of atrogin-1 mRNA was 30x higher at presentation than in normal controls, then normalized after adrenalectomy, and inversely correlated with LV ejection fraction [38]. In immunohistochemistry analysis, the expression of atrogin-1 was significantly higher in CS cardiomyopathy compared to patients with idiopathic cardiomyopathy and normal controls.

Links between glucocorticoids and this specific subtype of myocardial damage have to be deeply understood. A likely responsible actor is atrogin-1 protein, which is a E3 ligase present in skeletal, cardiac, and smooth muscle, able to downregulate myocardial protein during skeletal muscle differentiation [38]. Singh et al. showed that the overexpression of atrogin-1 increases the contractility of cultured smooth cell cultures and mouse aortic tissues in organ culture. Furthermore, atrogin-1 plays a pivotal role in the regulation of smooth muscle contractility through the enhancement of myocardin ubiquitylation/degradation and its transcriptional activity.

**Figure 2:**
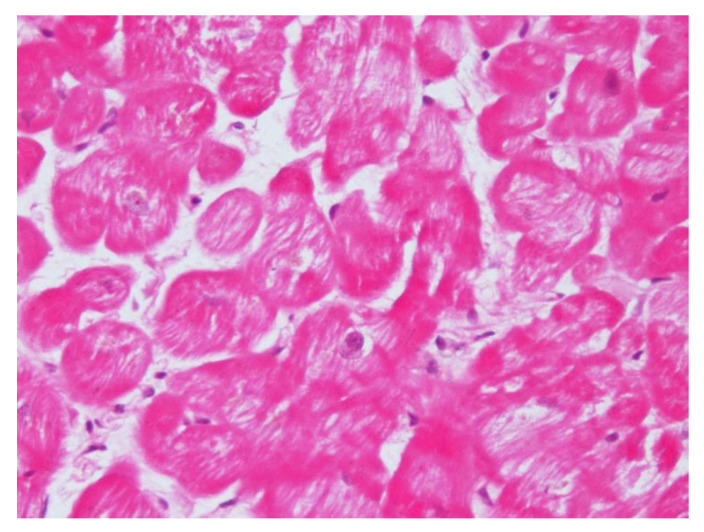
Hematoxylin and eosin staining (200×) shows a reduction in contractile elements because of gluconeogenesis due to cortisol overproduction in Cushing’s syndrome.

After their first observation, Frustaci et al., confirmed CS cardiomyopathy features in another eight patients with dilatative cardiomyopathy due to cortisol-secreting adrenal adenoma [39], concluding that CS cardiomyopathy shares in common the following features: cardiomyocyte hypertrophy, myofibrillolysis, and myocardial fibrosis (Figure 2). A pathophysiological link is involved in the pressure overload and glucocorticoid-mediated augmentation of angiotensin II that are jointly responsible for cell hypertrophy. Cardiomyocytes’ response to angiotensin II is enhanced. Cell myofibrillolysis is directly and strictly related to the enhanced myocardial expression of atrogin-1, as its expression was enhanced more than 38-fold during heart failure.

Increased plasma cortisol levels determine the activation of forkhead box (FOXO) transcription factors, with the subsequent activation of proteasome proteolysis, myofibrillolysis, and cell death [40]. Atrogin-1 returns to physiological levels after the treatment of cortisol excess and the restoration of cardiac damage, and it is also associated with a significant decrease in myofibrillolysis cell area from 61 to 22%. All these data confirm the effects of glucocorticoids on skeletal and cardiac muscle atrophy previously obtained in experimental models.

Microscopic and ultrastructural evidence is translated to phenotypic abnormalities of LV structure and function, reversible after the normalization of hypercortisolism. However, it has been shown that subjects may continue to exhibit exercise intolerance due to steroid-induced myopathy and following muscle weakness [41]. However, the treatment of CS usually results in the improvement or cure of hypertension, although hypertension could persist in patients with long-standing hypercortisolism and co-existing essential hypertension [42].

## 4. Catecholamine-Induced Cardiomyopathies

Pheochromocytoma (PHEO) is a rare neuroendocrine tumor arising from chromaffin cells [43], and originating from the sympathetic nervous system, which synthesizes and secretes catecholamines, adrenergic peptides, and their metabolites. The clinical presentation of PHEO is characterized by extreme variability. The most frequent symptoms reported are hypertension, palpitations, headaches, and diaphoresis [44].

We previously described our clinical experience of over 16 years [45]. In 91 patients with PHEO, 80 (88%) were generally symptomatic, and the classical triad (constituted by palpitations, headache, and diaphoresis) was present in 30% of patients affected by arterial hypertension, and less than 10% in normotensive subjects. Furthermore, 20% of patients were under medical treatment, while labile hypertension with paroxysmal attacks was present in 18% of cases. All these data, in agreement with the literature, confirmed the thesis that subjects with PHEO could range from paroxysmal or sustained hypertension to a normotensive profile. Therefore, it is extremely important to suspect a catecholamine-producing tumor in those patients also without classical symptoms.

It has been reported that PHEO is associated with many types of CMPs, including peripartum, hypertrophic, dilated, and Takotsubo-like. Several studies demonstrated that different systemic phenotypes depend on the duration and specific kind of catecholamine exposure [46].

Interestingly, Zhang et al., analyzed reviews of the literature from 1991 to November 2016, selecting 163 cases from 150 articles [47]. In overall case series, there were 163 cases presenting with PHEO and CMP, of whom 63 had dilated cardiomyopathy, 38 Takotsubo cardiomyopathy, 30 inverted Takotsubo cardiomyopathy, 10 hypertrophic cardiomyopathy, eight myocarditis, and 14 unspecified cardiomyopathies. Most cases lacked the classical phenotype of PHEO, and only 4% presented with the classical triad of headaches, palpitations, and diaphoresis.

This study was interesting because it was the first to highlight the atypical presentation of PHEO-induced cardiomyopathy, analyzing each subtype of cardiomyopathy associated with this condition.

One mechanism of myocardium damage related to catecholamine excess includes increased contractility, inducing functional hypoxia and, on the other hand, decreased blood flow, inducing coronary spasm. It is well documented that excessive free fatty acid-induced mitochondrial uncoupling increases oxygen consumption, as well as intracellular calcium excess and oxidative stress [47].

The abnormal stimulation of catecholamines exerts its detrimental effects in direct and indirect ways. The first one is characterized by the β1 adrenoreceptor transduction pathway, leading to calcium overload in cardiomyocytes [48]. During stressful situations, catecholamines generate indirect damage to the heart by oxygen radicals that cause coronary artery spasm, arrhythmias, and cardiac dysfunction. Moreover, the long-term spillover of catecholamines leads to the downregulation of β-adrenergic receptors, so the function of myofibers becomes suboptimal and contracting units are reduced [49].

In the literature, cardiovascular damage induced by sympatho-mimetic amines has been well described. Microscopically, the first insult is diffuse edema and a modest loss of striations in cardiomyocytes, and subsequently foci of myocardial cellular necrosis can be found [50]. Interestingly, in cocaine abuse, we observe similar mechanisms of the damage of myocardial toxicity, caused by an inappropriate endogenous hypercatecholaminergic secretion. These features include marked oxidative stress and mitochondrial dysfunction. The chronic use of cocaine determines various degrees of systolic and diastolic dysfunction, cardiac hypertrophy, and dilation [51]. Histologically, it is possible to observe the loss of myofibrils, multiple foci of band contractions, and fibrosis [52].

In Takotsubo cardiomyopathy, Lyon et al., suggested that excessive catecholamine stimulation could promote the expression of β2-receptors more prominently in the apex, leading to transient negative inotropism and hypokinesis, because Gs reverts signaling with a decreased stimulation [53]. In hypertrophic or dilated cardiomyopathy, stimulated myocytes induce adaptive mechanisms, including β-receptor internalization and subsequent desensitization. These modifications provoke interstitial fibrosis, myocyte apoptosis, and contractile dysfunction [48].

Moreover, Lassnig et al., described the case of a PHEO patient with Takotsubo-like apical cardiomyopathy, who developed CMP 6 months after a cardiogenic shock. Physicians must keep in mind that PHEO-associated CMPs may present with pulmonary edema or acute chest pain and myocardial ischemia/infarction. Pulmonary edema is the result of increased pulmonary capillary permeability, augmented peripheral vascular resistance, and hydrostatic pressures, inducing the final overfilling or constriction of the efferent pulmonary vein [54].

**Figure 3:**
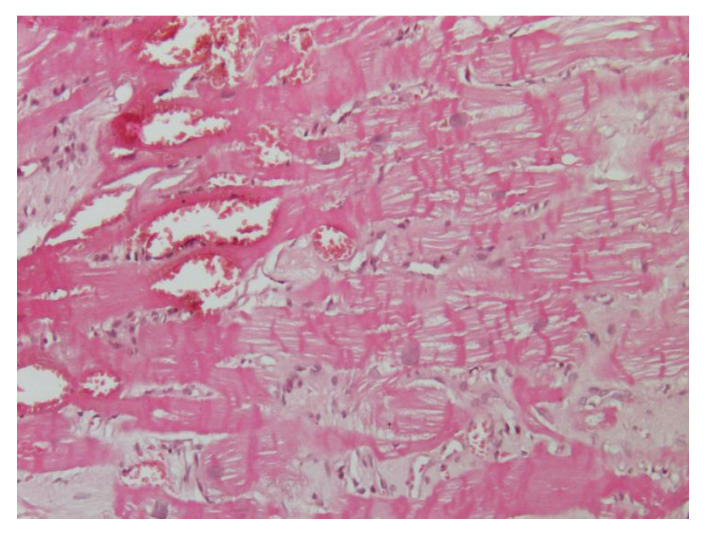
Hematoxylin and eosin staining (200×) shows the hypercontraction of sarcomeres with contraction band necrosis and lymphocytic infiltration due to catecholamine overproduction from pheochromocytoma.

We previously described a case report of a 37-year-old woman presenting with an history of dyspnea, chest pain, palpitation, and hypertension [55]. Electrocardiogram, echocardiogram, and cardiac magnetic resonance showed severe LVH with a prevalent involvement of the anterior portion of the interventricular septum. EMB demonstrated features of the hypercontraction of myofilaments with the formation of contraction bands and occasional cellular necrosis and wide and perivascular fibrosis. Electron microscopy at higher magnifications showed the catecholamine-induced diffuse contraction with band necrosis. Finally, we found a rare cases of catecholamine-induced CMPs due to adrenal adenoma mixed with nodules enriched in epinephrine-type secreting granules (Figure 3).

Interestingly, most authors agree that catecholamine-induced CMP improves after the surgical eradication of PHEO. The reversal of cardiomyopathy depends on early detection and intervention. However, in the case of acute heart failure and significant myocardial damage, prognosis is very poor [56].

## 5. Conclusions

Cardiomyopathies due to adrenal hormone excess are interesting clinical entities. Previously, a coexistent cardiomyopathy associated with mineralocorticoid, glucocorticoid, or amine excess was not clearly explained, though the detrimental effects derived from the oversecretion of these hormones are well known and produce profound remodeling in the myocardium. However, the early detection and treatment of the disorders may determine reverse cardiac remodeling.

Pivotal insights into the pathophysiology of these peculiar cardiomyopathies are provided by EMB, followed by histologic, immunohistochemical, and molecular biology analysis.

The newest microscopic and ultrastructural evidence is precious and irreplaceable to understand the underlying physiopathology through which these hormones determine pathway activation and then the structural alteration of cardiomyocytes.

Indeed, vacuolar degeneration and edema in PA, multiple foci myofibrillolysis in CS, and contraction band necrosis in PHEO are histological features that are peculiar and unique.

EMB has been shown to be a safe and effective tool in diagnosing structural substrate abnormalities in the course of several cardiomyopathies and may have a key role in the understanding, diagnosis, and management of adrenal-related cardiac disease.

This literature review, and the exhibition of brand new macro- and microscopical cardiac features involved in these pathologies, may allow physicians to better understand the underlying mechanisms of these forms of arterial hypertension sustained by adrenal hormone excess.
